# Bacillus Calmette Guerin Induces Fibroblast Activation Both Directly and through Macrophages in a Mouse Bladder Cancer Model

**DOI:** 10.1371/journal.pone.0013571

**Published:** 2010-10-22

**Authors:** Catalina Lodillinsky, Yanina Langle, Ariel Guionet, Adrián Góngora, Alberto Baldi, Eduardo O. Sandes, Alberto Casabé, Ana María Eiján

**Affiliations:** 1 Research Area, Institute of Oncology Angel H. Roffo, University of Buenos Aires, Buenos Aires, Argentina; 2 Molecular Pathology and Pharmacology Laboratory, Institute of Biology and Experimental Medicine (IBYME)-National Research Council of Argentina (CONICET), Buenos Aires, Argentina; Institut Pasteur, France

## Abstract

**Background:**

Bacillus Calmette-Guerin (BCG) is the most effective treatment for non-muscle invasive bladder cancer. However, a failure in the initial response or relapse within the first five years of treatment has been observed in 20% of patients. We have previously observed that *in vivo* administration of an inhibitor of nitric oxide improved the response to BCG of bladder tumor bearing mice. It was described that this effect was due to a replacement of tumor tissue by collagen depots. The aim of the present work was to clarify the mechanism involved in this process.

**Methodology/Principal Findings:**

We demonstrated that BCG induces NIH-3T3 fibroblast proliferation by activating the MAPK and PI3K signaling pathways and also differentiation determined by alpha-smooth muscle actin (alpha-SMA) expression. *In vivo*, intratumoral inoculation of BCG also increased alpha-SMA and collagen expression. Oral administration of L-NAME enhanced the pro-fibrotic effect of BCG. Peritoneal macrophages obtained from MB49 tumor-bearing mice treated *in vivo* with combined treatment of BCG with L-NAME also enhanced fibroblast proliferation. We observed that FGF-2 is one of the factors released by BCG-activated macrophages that is able to induce fibroblast proliferation. The involvement of FGF-2 was evidenced using an anti-FGF2 antibody. At the same time, this macrophage population improved wound healing rate in normal mice and FGF-2 expression was also increased in these wounds.

**Conclusions/Significance:**

Our findings suggest that fibroblasts are targeted by BCG both directly and through activated macrophages in an immunotherapy context of a bladder murine model. We also described, for the first time, that FGF-2 is involved in a dialog between fibroblasts and macrophages induced after BCG treatment. The fact that L-NAME administration improves the BCG effect on fibroblasts, NO inhibition, might represent a new approach to add to the conventional BCG therapy.

## Introduction

At the time of diagnosis, 60–80% of bladder tumors are non-muscle invasive and confined to the urothelium and/or lamina propria. These include papillary tumors or carcinoma in situ. Both types of tumors commonly occur concurrently. In 1976, Morales et al. [1] reported, for the first time, the successful intravesical use of *Bacillus Calmette Guerin (BCG)* as an adjuvant treatment for non-muscle invasive bladder cancer following transurethral resection. It is now widely accepted that intravesical BCG is more potent therapy in preventing tumor recurrence than any intravesical chemotherapy [2]. However, about 20% of patients either fail to respond initially or relapse within the first five years of treatment [3].

It is known that BCG generates a local immunological reaction with activation of immune cells as well as secretion of cytokines involving Th1 cell cytotoxicity [4]. A significant increase in polymorphonuclear and mononuclear cell that infiltrate in bladder tumors after BCG therapy has been observed [5]. Since macrophages (MACs) are phagocytic and antigen presenting cells and have the capacity to secrete cytokines and growth factors, they are considered the best equipped cells involved in BCG immunotherapy. Depending on the microenvironment, the nature and intensity where MACs differentiation takes place, these cells are able to activate different pathways and give rise to particular profiles [6]. The responses of MACs following injury or infection are examples of many different stimuli that trigger MACs activation in tissues, displaying great plasticity. BCG, when used as immunotherapy for bladder tumors, is processed by MACs and urothelial cells, resulting in the early release of inflammatory cytokines, some of which may be responsible for certain adverse effects observed in patients [7], [8]. One of the mediators of this inflammatory process is nitric oxide (NO), generated by a family of NO synthases (NOSs). Inflammatory cytokines and/or bacterial products usually activate the expression of the inducible NOS (iNOS) isoform, generating large amounts of NO. iNOS is not expressed in normal bladder epithelium but has been detected in early bladder tumor recurrences [9] and it has been reported that iNOS expression in tumor cells could be associated with unresponsiveness to BCG [10]. We have previously reported that in vivo administration of BCG to MB49 tumor bearing mice decreased tumor growth and that the combined treatment of BCG with the NOS inhibitor L-NAME significantly improved tumor regression by replacing tumor tissue by collagen depots, resembling wound healing [11]. Our present results suggest that control of bladder tumor recurrences by BCG therapy involve stroma reorganization and that NO inhibition might improve tissue remodeling. Wound healing is an example of tissue reorganization, since after wound generation, growth factors released to the extracellular matrix induces an inflammatory process which allows cell migration [12]. Among others, MACs and fibroblast are important cells involved in this process. Fibroblast migrate towards the damaged zone, differentiate into myofibroblasts and synthesize extracellular matrix proteins that allow the contraction and finally the wound close. In a wound healing context, growth factors such a fibloblast growth factor-2 (FGF-2) and transforming growth factor beta (TGF-beta) secreted by MACs, stimulate fibroblasts which are responsible for the synthesis, deposition and remodeling of the extracellular matrix [13]. FGF-2 was originally identified as a basic growth factor which stimulates the proliferation of NIH-3T3 fibroblasts. Besides, several studies have shown a role of FGF-2 in tissue fibrosis, where this factor is increased in acute wounds and plays a role in granulation tissue formation, reepithelization and tissue remodeling [12], [14].

To our knowledge, there is no information about the role in tissue remodeling of the BCG when used in bladder cancer treatment. Therefore, the aim of our work was to evaluate the effect of BCG on fibroblast activation, measured by MAPK and PI3K signaling pathways and collagen I, and alpha-smooth muscle actin (alpha-SMA) expression. Since MACs are involved in both BCG response and in tissue reorganization as observed in the process of wound healing [6], [15] we also evaluated the role of MACs under BCG treatment and NO inhibition therapy, in fibroblast activation in a wound healing model. Our findings suggest that, as part of the mechanisms of bladder cancer control, BCG induces activation of fibroblasts either directly or through MACs, that by releasing soluble products, can by themselves activate fibroblasts. The participation of NO as a negative regulator of this process was also demonstrated.

## Results

### BCG induces NIH-3T3 proliferation

**Figure 1:**
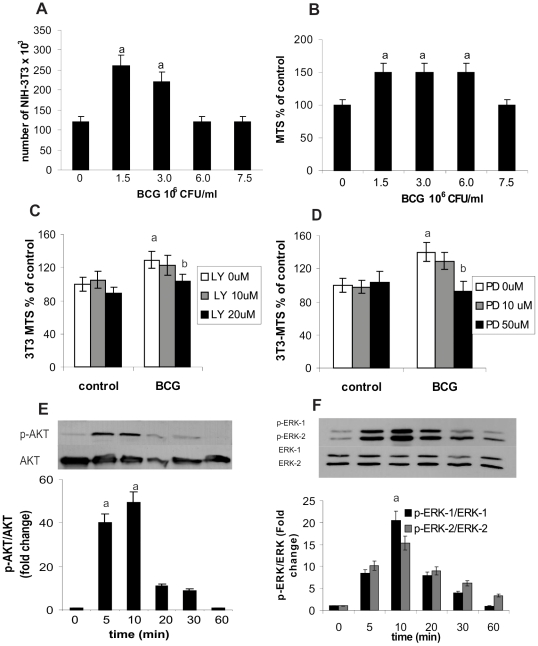
BCG induces fibroblast proliferation. (A) NIH-3T3 fibroblast were treated with different concentration of BCG for 24 h. Cells were counted or (B) cell viability was evaluated by a non-radioactive cell titter method (MTS), vs control: p<0.05. (C) Effect of LY 294002 and (D) PD 98059 evaluated by MTS in fibroblast stimulated with BCG (3×10^6^ CFU/ml) for 24 and 48 h respectively, a: p<0.05 vs control, b: vs BCG. Fibroblast stimulated with BCG (3×10^6^ CFU/ml) and p-AKT (E) and p-ERK (F) determined by Western blot. Relative phosphorylation levels were normalized to total protein and referred as a fold change of control, a: p<0.05.

It has been described that BCG is able to induce cell cycle arrest and apoptosis in bladder tumor cell lines [15], [16]. However, one remaining question could be what would be the effect of BCG on fibroblasts? To answer this question NIH-3T3 cells were cultured under different concentrations of BCG. As shown in Fig. 1A and 1B, BCG induced the proliferation of NIH-3T3 cells, evaluated both by counting the number of cells and by the MTS assay. Induction of fibroblast proliferation was detected with BCG concentrations from 1.5×10^6^ CFU/ml up to 3×10^6^ CFU/ml, diminishing for concentrations higher than 6×10^6^ CFU/ml. Our results show discrepancies when the determination of the proliferation was made either by counting the number of cells or by MTS (figure 1 A and B respectively) for concentrations equal or greater than 6×10^6^ CFU/ml. We think that this difference was related to enhance of mitochondrial activity induced by high quantities of BCG. Thus, the following experiments were carried out with 3×10^6^ CFU/ml of BCG for which there is agreement between the two forms of quantification. To evaluate whether PI3K and MAPK pathways are involved in BCG-induced fibroblast proliferation, we analyzed the effects of LY 294002 and PD 98059, inhibitors of PI3K and MAPK pathways respectively, on fibroblast proliferation induced by BCG. We also evaluated the phosphorylation of ERK and AKT, two activated molecules that are key in these pathways. We observed that LY (20 µM) was able to inhibit the proliferation induced by BCG after 24 h of treatment (Fig. 1C), and that PD (50 µM) inhibited the proliferation induced by BCG after 48 h of treatment (Fig. 1D). As shown in Fig. 1E and 1F, BCG treatment induced a rapid phosphorylation of ERK and AKT within 5 min. Western blot analyses revealed that the phosphorylation of AKT was stimulated between 5–10 min, decreasing after 20 min (Fig. 1E). BCG also induced ERK1 and ERK2 phosphorylation after 5 min, which then diminished after 30 min (Fig. 1F). This interaction between BCG and fibroblast involves both a proliferation and a survival pathway. These data suggest that BCG targets not only tumor cells and MACs, but also fibroblasts.

**Figure 2:**
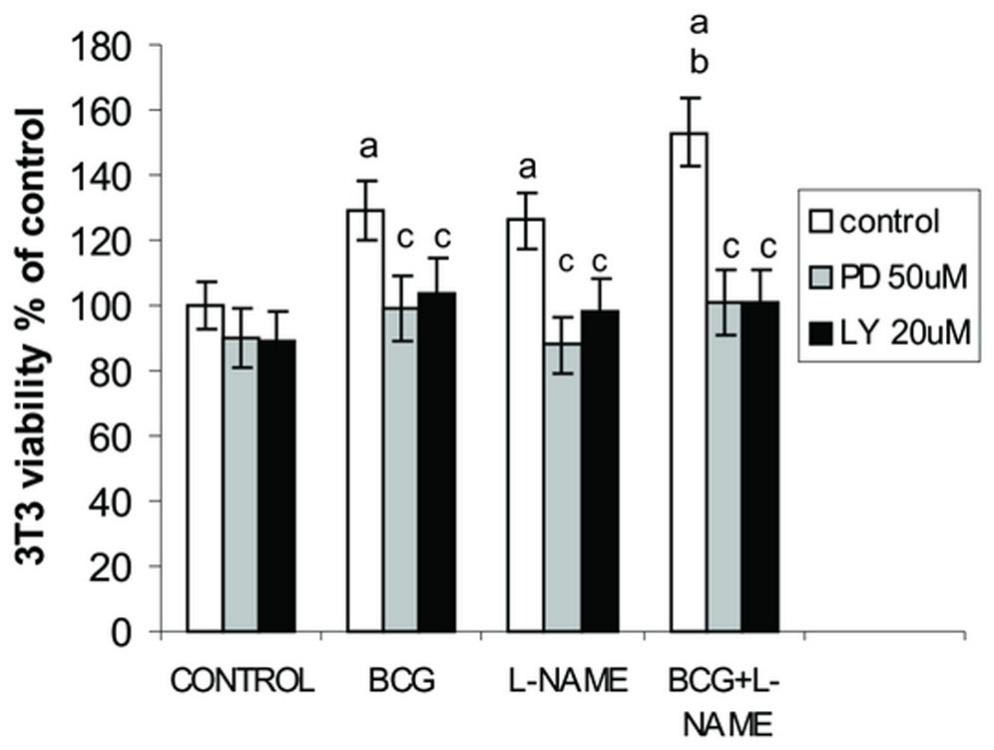
L-NAME improves BCG-induced proliferation. NIH-3T3 fibroblast were treated with BCG (3×10^6^ CFU/ml), L-NAME (2 mM) or BCG and L-NAME in presence of LY 294002 and PD 98059 for 24 and 48 h respectively. Cell viability was evaluated by MTS. a: p<0.01 vs Control; b: p<0.05 vs BCG or L-NAME alone; c:p<0.01 vs their respective Control.

The proliferation of NHI-3T3 cells was enhanced by L-NAME from 0.5 to 4 mM (p<0.05) in a concentration-independent manner (data not shown). When fibroblasts were treated with a combined protocol, the highest proliferation activity was detected with BCG 3×10^6^ CFU/ml plus L-NAME 2mM (Fig 2). This effect was inhibited by LY 294002 and PD 98059, thus indicating that L-NAME is able to improve BCG-induced proliferation in NIH-3T3 cells mediated by MAPK and PI3K signaling pathways.

### BCG induces NIH-3T3 differentiation

Activation of fibroblasts into myofibroblasts, a crucial step in the process of wound healing, is characterized by the development of intracytoplasmic stress fibers that confer to these cells the capacity of developing tension, and by the increased synthesis of extracellular matrix components, such as collagen type I [17], [18]. The most important marker of the fibroblast/myofibroblast phenotypic transition is the novo expresión of alpha-smooth muscle actin [19]. So, we analyzed alpha-SMA and collagen I as indicators of fibrogenic activity and evaluated the expression of these molecules in NIH-3T3 cells treated with BCG.

**Figure 3:**
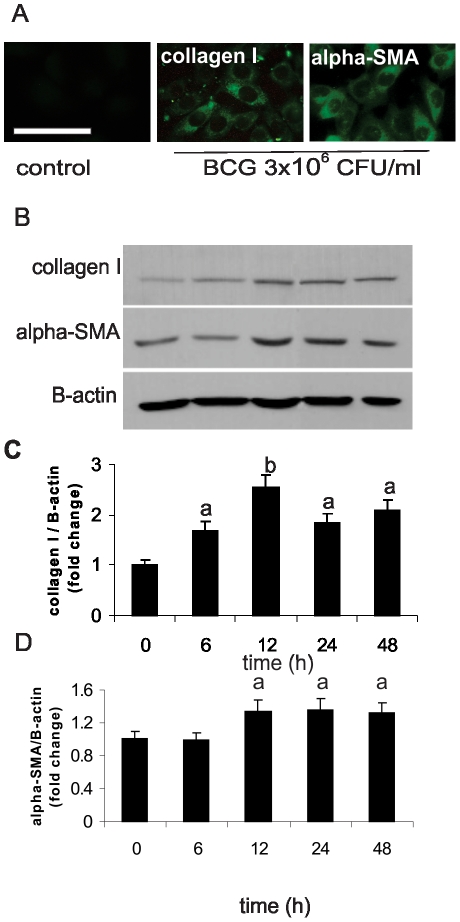
BCG induces fibroblast differentiation. (A) Immunofluoresce staining of NIH-3T3 treated with BCG (3×10^6^ CFU/ml) for 24 h revealed with anti-collagen I and anti-alpha-SMA antibody. Scale: bar = 100 um. (B) Western Blot from fibroblast homogenates treated with BCG (3×10^6^ CFU/ml) at different times to determinate collagen I and alpha-SMA induction. (C) Densitometric units of collagen I or (D) alpha-SMA were determined using analysis software, relativized to beta-actin and referred as a fold change of control a: p<0.05, b: p<0.01.

To establish the best dose of BCG, immunofluorescence of both proteins was carried out in fibroblasts treated with different concentrations of BCG for 24 h (data not shown). The strongest expression of both alpha-SMA and collagen I was observed with 3×10^6^ CFU/ml of BCG (Fig. 3A), thus, this concentration was chosen to carry out the Western blots analyses. Figure 3B shows that 3×10^6^ CFU/ml of BCG was able to induce collagen I expression after 6 h of treatment, being the expression 2.5-fold higher than controls after 12 h of treatment. A significant induction of alpha-SMA after 12 h of BCG treatment was observed, remaining increased at 48 h post treatment. Taken together the data showed up to now, we could hypothesize that fibroblast activation can also take place in vivo.

### BCG induces fibroblast activation through macrophages *in vitro* and induces collagen and alpha-SMA expression in MB49 tumors *in vivo*

**Figure 4:**
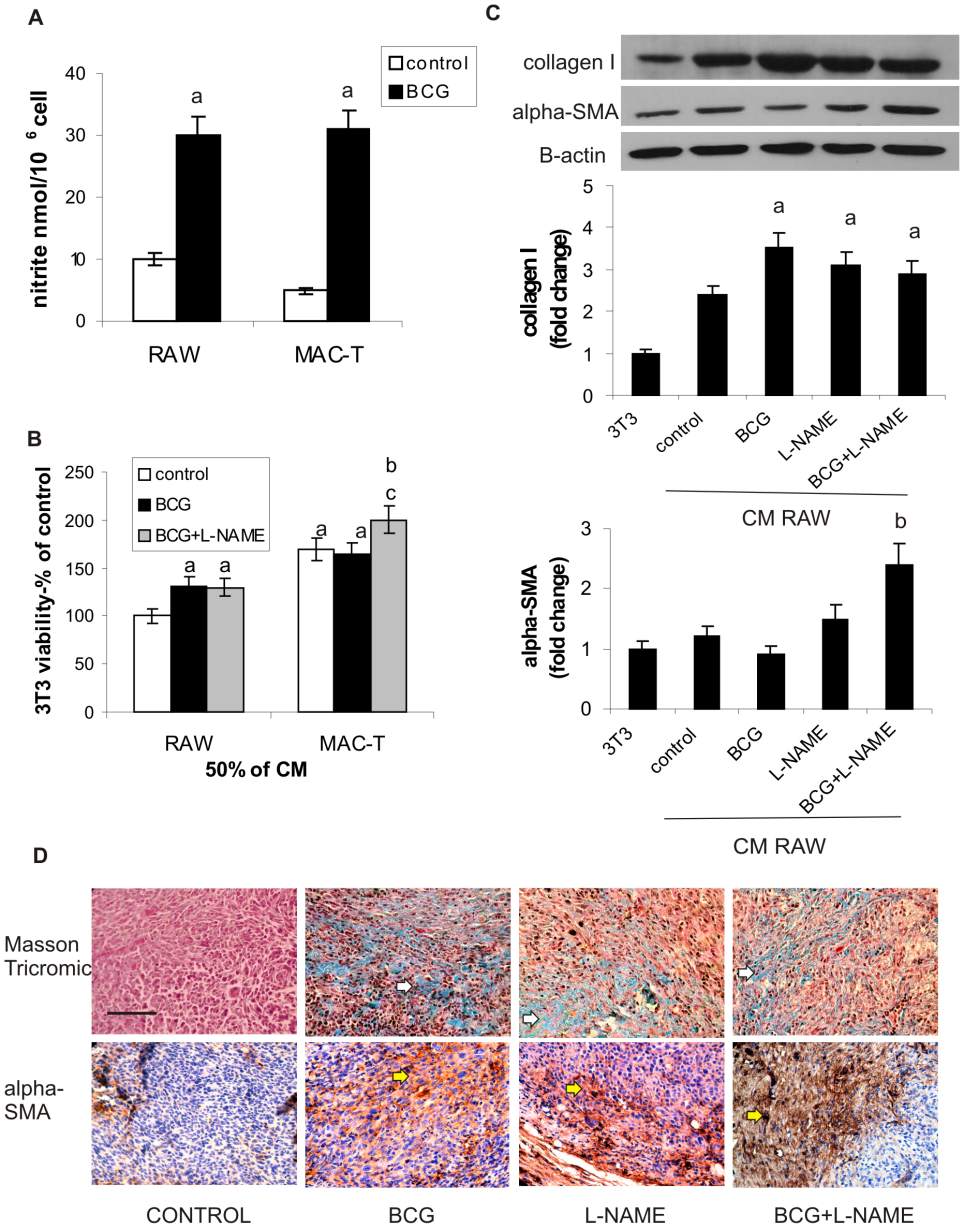
BCG induces fibroblast activation through macrophages. (A) NO production was determined in the supernatants of both peritoneal MACs from MB49 tumor bearing mice (MACs-T) and RAW 264.7 cells treated in vivo with BCG (6×10^6^ CFU/ml) or in vitro (3×10^6^ CFU/ml) respectively, were evaluated in the supernatant by Griess reagent. a: p<0.0001 vs control. RAW 264.7 cells were treated with BCG (3×10^6^ CFU/ml) for 24 h, and then cells were extensively washed with PBS. Serum-free medium was added and incubation was continued for 24 hours to obtain the CM. (B) fibroblast treated for 48 h with the CM from peritoneal or RAW 264.7 cells previously treated with BCG (3×10^6^ CFU/ml) plus L-NAME (2 mM). Fibroblast viability was evaluated by MTS and referred as a percentage of control (RAW 264.7 or NIH-3T3 untreated cell lines), a: p<0.05 and b: p<0.01 vs control, c: p<0.05 vs MACs-T control. (C) Western Blot to determine collagen I and alpha-SMA from fibroblast homogenates treated for 24 h with CM from RAW 264.7 previously treated for 24 h with BCG plus L-NAME. Relative expression level was normalized to beta-actin and referred as a fold change of control, a: p<0.05, b: p<0.01. (D) Masson Trichome (top panel) and immunohistochemical staining (bottom panel), to determine collagen fibers and alpha-SMA respectively, were performed in MB49 tumors growing subcutaneously in mice treated with BCG, L-NAME or BCG+L-NAME. White arrows show blue stain of collagen fibers. Yellow arrows show brown positive staining for alpha-SMA. Scale: bar = 100 um.

As the direct treatment with BCG induces fibroblast proliferation and MACs are some of the most important cells in BCG responses, we hypothesized that there is a dialog between fibroblasts and MACs under BCG treatment. In order to test this, we investigated whether MACs treated with BCG can affect the proliferation and differentiation of fibroblasts. First, we evaluated NO production in MACs treated with BCG. As shown in Fig. 4A, peritoneal MACs from tumor-bearing mice treated in vivo with BCG produced higher amounts of NO than those from non-treated ones. In vitro, the treatment of the MACs cell line RAW 264.7 with BCG also increased NO production. To evaluate the role of the soluble products released from MACs, we obtained the conditioned media (CM) from peritoneal MACs from tumor-bearing mice either treated in vivo or not with BCG +/− L-NAME. We observed that CM from peritoneal MACs from tumor-bearing mice (MACs-T) of the different groups induced fibroblast proliferation. Particularly, the CM of MACs from tumor-bearing mice treated in vivo with BCG (MACs-T-BCG) plus L-NAME induced the highest fibroblasts proliferation rate in vitro. The CM from untreated RAW 264.7 did not modify fibroblast proliferation. However, the in vitro treatment of RAW 264.7 with BCG either combined or not with L-NAME, similarly to peritoneal MACs, also induced NIH-3T3 proliferation (Fig. 4B).

The CM obtained from RAW 264.7 induced collagen I expression in fibroblasts. The expression was higher when the CM was from MACs treated with BCG, and this effect was remained high under L-NAME addition. The CM obtained from RAW 264.7 treated with BCG plus L-NAME also induced alpha-SMA expression (Fig. 4C). These results seem to indicate that there are some soluble factors secreted from MACs activated by BCG which can induce fibroblast proliferation and activation. With the aim to evaluate this effect in vivo we analyzed collagen deposition and alpha-SMA expression in MB49 tumor-bearing mice under BCG and L-NAME treatment. Our results showed that both BCG and L-NAME and their combination induced the deposition of collagen fibers in these tumors. BCG and L-NAME also induced expression of alpha-SMA compared with the control group. However, more intense staining of alpha-SMA was observed in tumors treated with BCG plus L-NAME. These results suggest that BCG may induce the activation of fibroblasts in vivo, and that this effect is enhanced by inhibition of NO production.

### Nitric oxide inhibition improves *in vivo* wound healing induced by peritoneal MACs from tumor-bearing mice treated with BCG

**Figure 5:**
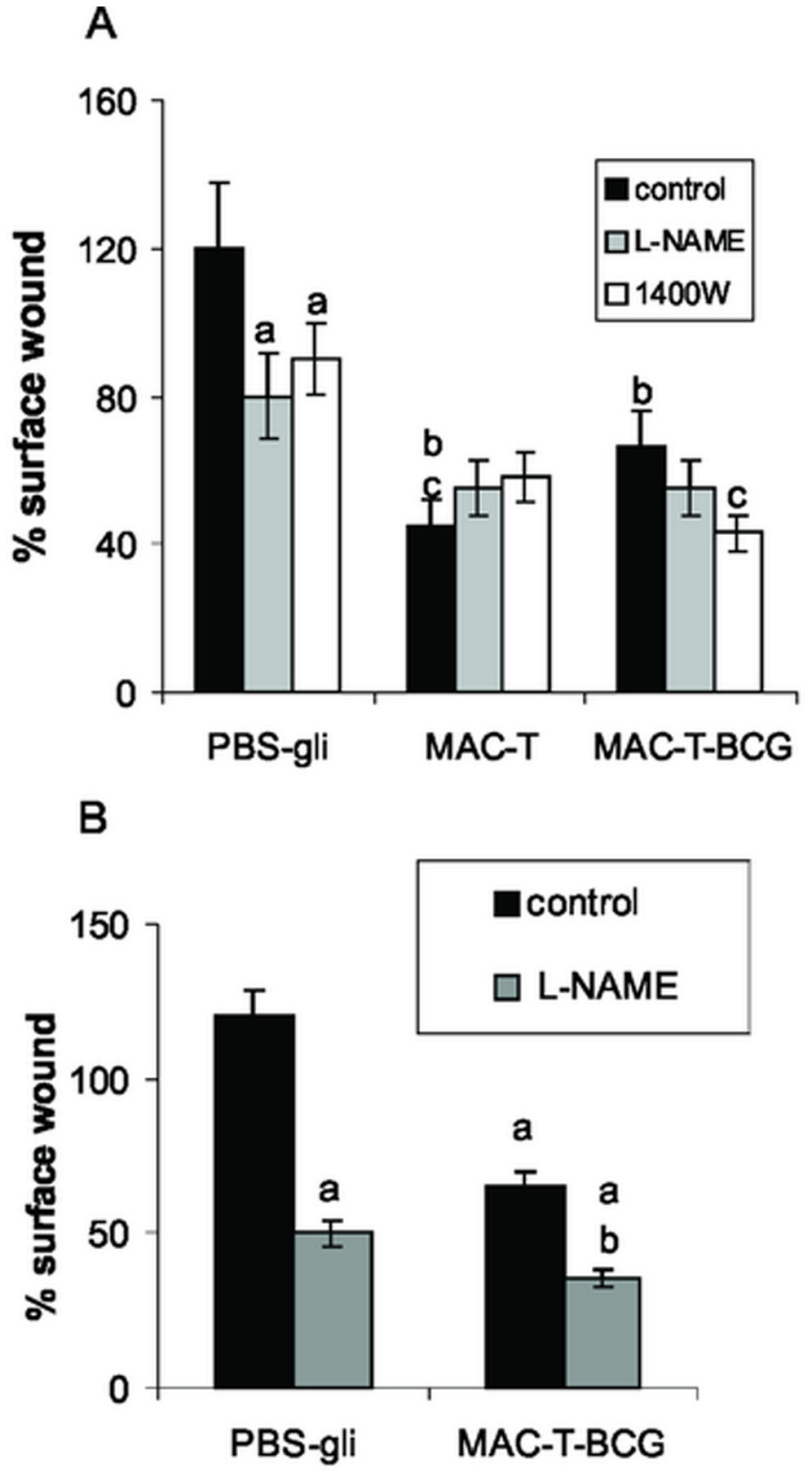
NO inhibition increases wound healing closure generated by peritoneal MACs. (A) Peritoneal MACs from tumor bearing mice either treated or not with BCG (MACs-T-BCG and MACs-T respectively), were placed in dorsal skin wound of normal mice. L-NAME (2 mM) or 1400 W (1 uM) was added onto the wound. The wound closure was calculated as the percentage of the initial wound area (day zero), a: p<0.01, b: p<0.001 vs PBS-gli, c: p<0.05 vs MACs-T-BCG control. (B) Peritoneal MACs-T-BCG were placed in dorsal skin wound of mice treated orally with L-NAME (0.2 g/kg mouse), a: p<0.0001 vs PBS-gli, b: p<0.01 vs MAC-T-BCG control.

To determine the functional capacity of MACs under BCG treatment and its regulation by NO, an in vivo experiment of wound healing was performed. Peritoneal MACs–T either treated or not with BCG in vivo were placed in a dorsal skin wound of normal mice. The NO inhibitors L-NAME and 1400W were added in wounds either alone or combined with MACs. The surface wound was significantly diminished when the MACs from normal (data not shown) or MACs-T were added on the wound as compared with the controls, where only PBS-glycerol solution was added. It is important to note that the addition of the NO inhibitors L-NAME or 1400W alone were able to significantly diminish the surface wound. When peritoneal MACs from tumor-bearing mice which had received BCG intratumorally (MACs-T-BCG) were added on the wound, the percentage of wound closure was diminished, as compared with the wounds with MACs-T. However, when these MACs-T-BCG were added with 1400 W, the wound healing rate was significantly increased (Fig. 5A).

When the dorsal skin wound was generated in mice drinking the NO inhibitor L-NAME, the surface wound was diminished compared with the control group. MACs-T-BCG addition also induced the acceleration of wound repair. Besides, MACs-T-BCG addition improved the wound repair when mice received L-NAME orally (Fig. 5B).

### FGF-2 mediates the stimulatory effect of BCG-activated macrophages on fibroblasts

**Figure 6:**
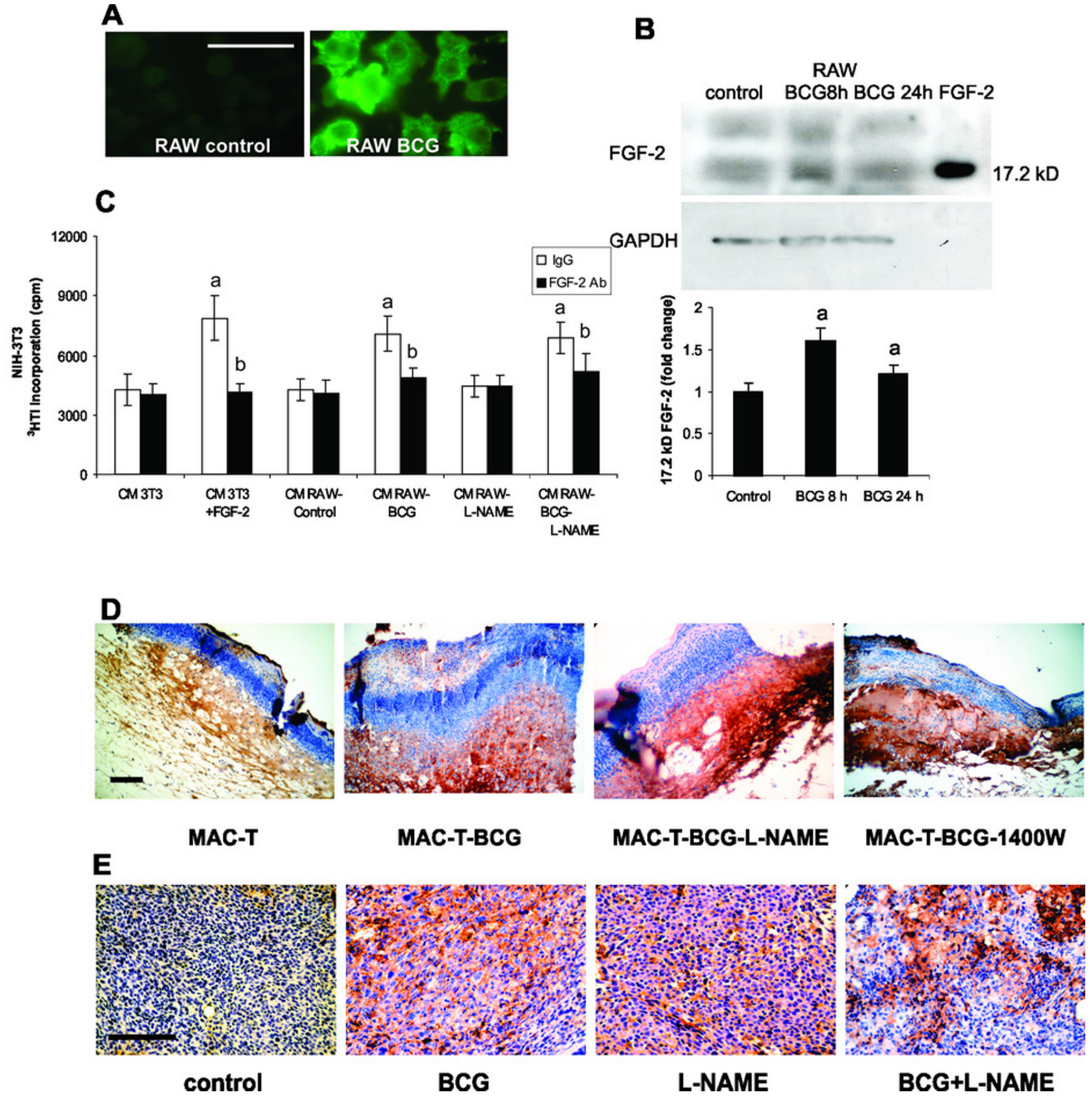
FGF-2 secreted by BCG-treated MACs induces NIH-3T3 cells proliferation. (A) Immunofluorescence staining with anti-FGF-2 antibody of RAW 264.7 MACs treated with BCG (3×10^6^ CFU/ml) for 24 h. (B) Western blot from lysates of RAW 264.7 treated with BCG (3×10^6^ CFU/ml) for 8 and 24 h. 20 ng of purified murine FGF-2 was used as a positive control. Relative expression level was normalized to GAPDH and referred as a fold change of control, a: p<0.05 (C) fibroblasts were treated with CM from RAW 264.7 cells previously treated with BCG (3×10^6^ CFU/ml), L-NAME or BCG plus L-NAME for 24 h. Control was carried out with exhausted culture media (CM 3T3, from the same NIH-3T3 cells). 0.5 ng/ml of FGF-2 in CM 3T3 was used as a positive control. NIH-3T3 proliferation was monitored by ^3^(H) thymidine incorporation. The CM was preincubated 1 h with 10 µg/ml of the blocking monoclonal anti FGF-2 antibody (DB3), or normal IgG as a control, a: p<0.001 vs CM NIH-3T3, b: p<0.001 vs IgG. (D) Immunohistochemical staining to determinate FGF-2 expression was performed in skin wounds. Wounds were treated with peritoneal MACs from tumor bearing mice treated or not with BCG (MAC-T and MAC-T-BCG respectivesly) alone or locally combined with NO inhibitors (E). Immunohistochemical staining of FGF-2 was performed in s.c. MB49 tumors from mice treated with BCG, L-NAME or BCG plus L-NAME. Scale: bar = 100 um.

It has been demonstrated that FGF-2 is one of the main growth factors involved in fibroblast proliferation [20]. Besides, it has been shown that FGF-2 is able to inhibit apoptosis in NIH-3T3 cells treated with chemotherapy drugs [21]. Thus we have hypothesized that FGF-2 could be one of the soluble factors secreted by activated MACs able to stimulate the proliferation of fibroblasts. To confirm this idea, we first analyzed whether FGF-2 is modulated in MACs RAW 264.7 cells under BCG treatment. Figure 6A shows, by Immunofluorescence staining, that FGF-2 is increased in MACs RAW 264.7 treated with BCG compared with untreated cells. This was also observed by western blot assay (figure 6B), where it can be seen one band of 17.2 kDa compatibly with secretory FGF-2. Bands of higher molecular weight represent non-secretory FGF-2. We then performed a proliferation assay with CM derived from RAW 264.7 activated by BCG, depleted or not of FGF-2. CM from RAW 264.7 treated with BCG increased fibroblast proliferation, while the depletion of FGF-2 significantly reduced the fibroblast stimulation, suggesting that BCG-activated MACs could induce fibroblast proliferation, at least in part, by FGF-2 secretion. Fibroblasts migrate into the wound, where they proliferate and produce large amounts of extracellular matrix. Some fibroblasts differentiante into myofibroblasts, which are responsible for the wound contraction and the deposition of additional matrix [22]. To evaluate if MACs from tumor bearing mice treated with BCG are capable to induce FGF-2 in vivo, expression of FGF-2 was evaluated either in wound healing assays. Beside, we also evaluated the FGF-2 expression in MB49 tumors. Histological analyses from skin wounds showed increased expression of FGF-2 in wounds with MACs-T-BCG. When these MACs were placed together with NO inhibitors on wounds, the FGF-2 expression remained high (Fig. 6D). The high level of FGF-2 was consistent with the increased healing rate that was showed in Fig. 5. Furthermore, similar results were observed in MB49 tumors, where the expression of FGF-2 was higher in tumors from mice treated either with BCG or with L-NAME than in controls. Tumors derived from mice receiving the combined treatment showed more localized and intensive FGF-2 staining (Fig. 6E). In this model, our results show that the FGF-2 is associated with mechanism of action BCG immunotherapy.

## Discussion

Intravesical therapy with BCG plays a major role in the treatment and prophylaxis of recurrent non-muscle invasive bladder carcinoma [23].

Shelley et al. [24] have recently described in a meta-analyses study that BCG used as adjuvant after transurethral resection reduced the risk of recurrence by 67% at 12 months compared to transurethral resection alone; however, some side effects such as cystitis, fever and hematuria, were associated with BCG administration. The exact mechanism of the antitumor activity of BCG is not completely understood, but it appears that BCG involves both direct effects on tumor cells [16] and indirect effects mediated by immune cells [7].

We have previously reported that BCG wass able to induce growth inhibition of MB49 bladder tumor cells either in vitro or inoculated vivo into syngeneic mice NO produced by MB49 cancer cells treated with BCG induced MACs and splenocytes death, thus suggesting an in vivo immunosuppressive function of NO. Our experiments also showed a greater inhibition of tumor growth in mice treated with BCG combined with L-NAME compared to BCG alone. In the first case, few remaining tumor cells were completely surrounded by collagen fibers [11].

In the present work, we investigate some of the mechanisms by which BCG and L-NAME generate the scar we described previously. The MAPK and PI3K signaling pathways have been widely studied in different models. These are activated by different stimuli such as growth factors and cytokines [25], [26]. Our present findings demonstrate that BCG is able to directly induce NIH-3T3 fibroblast proliferation through MAPK and PI3K signaling pathways, collagen expression and fibroblast differentiation as determined by alpha-SMA expression. Besides, immunohistochemistry of tumors from BCG treated mice, showed increased expression of collagen fibers and alpha-SMA, suggesting that fibroblast activation could also take place.

We believe that BCG can act on the stroma surrounding tumor cells that may remain after tumor resection or on a new rising tumor. Thus, the joint activity of fibroblast and immune cells activated by BCG and the direct action on tumor cells would reduce the risk of recurrence. The concentration of BCG used in each instillation in patients, is approximately of 10^7^ CFU/ml (total volume 50 ml). We do not know the exact dosis that receive the stroma, nevertheless, in our experiments we use 3×10^6^ CFU/ml, which is the best dosis that induces the fibroblast proliferaton in vitro, being one lower order than that used in the instillation of patient. Thus, we speculate that the quantities of BCG we employ in vitro are almost physiological.

It is known that bacterial stimuli are able to induce NO production in MACs [27]. Here we showed that after BCG treatment, RAW 264.7 and MACs-T were able to increase NO levels in vitro and in vivo respectively and CM from RAW 264.7 treated in vitro with BCG induced fibroblast proliferation and increased collagen I expression. Because of the involvement of NO in various aspects of physiological and pathological processes, NOS inhibitors have gained prominence in the mechanisms involved in wound healing, angiogenesis and inflammatory response to cytokines [28], [29], [30]. Since CM from MACs-T-BCG-L-NAME induced the highest fibroblast proliferation rate, we could speculate that in this cell population, inhibition of NO induces the release of some soluble factors, able to increase fibroblast proliferation and activation. Besides, we cannot discard that NO inhibition may also modify the secretion pattern of these soluble factors. To our knowledge, there are no reports showing the interaction between MACs and fibroblast in bladder cancer. However, in concordance with our results, using a pulmonary tuberculosis model, it has been described enhanced proliferation of pulmonary fibroblast by CM of alveolar macrophages stimulated with BCG [31]. Concurrently, we observed concordance between the effect of the in vivo treatment of bladder tumos and the in vitro assay. A marked expression of collagen fibers and alpha-SMA was observed in tumors treated with BCG, L-NAME and BCG plus L-NAME, being the effect more pronounced with the combined treatment.

The role of NO in dermal fibroblast proliferation has been previously described by Chen et al [32], who suggested that inhibition of dermal fibroblast proliferation by UV light, might be related to the up-regulation of iNOS gene expression and thus to NO over-secretion In agree with these results, we may suggest that NO production is a negative regulator of fibroblast proliferation and activation when BCG is used in bladder cancer therapy.

Tumors and wounds share some components, such as the inflammatory context [22]. However, while in wounds inflammation is transient, in tumors this process is perpetuated in time, making this reversion, a goal for tumor growth control. In 1986, Dvorak postulated the concept of “tumors are wounds that do not heal” and hypothesized that the composition of the tumor stroma resembles the granulation tissue of healing skin wound, which suggests that epithelial tumors promote the formation of their stroma by activating the wound healing response of the host [22], [33]. Based on this hypothesis, we generated a dorsal skin wound assay, to evaluate the role of macrophages under different treatments in a model where fibroblasts have a key function. We evaluated the capacity of MACs-T-BCG to help in wound healing. We observed that exogenous MACs-T accelerated the healing process, but on the contrary MACs-T-BCG decreased healing rate compared with MACs-T. However, in the presence of a NO inhibitor, the wound closure was accelerated, suggesting that the NO released by MACs-T-BCG delays the healing process. To evaluate the role of endogenous NO of the host, L-NAME was orally administered to wound-bearing mice, the wound closure was significantly faster when MACs-T-BCG were placed in the wounds. In summary, our results suggest that, besides the NO released onto the wound by MACs-T-BCG, other cells producing NO in wound-bearing mice play a negative role in the healing process. The information related to NO activity in wound repair is controversial. Some authors have shown that NO induces an angiogenic process necessary for a good healing [29], [34], whereas others have demonstrated that apoptosis induced by NO is able to inhibit wound healing [30]. On the basis of our results, we may speculate that the NO generated by MACs-T-BCG or by the remaining cancer cells can delay the tissue reorganization during the BCG immunotherapy.

Divergent functions of growth factors, such as FGF-2 or TGF-beta, in wound healing and cancer have also been reported. It is well known that during the wound healing process, MACs secrete FGF-2 and TGF-beta1 which influence fibroblast proliferation [13]. Since in our model we observed that the RAW 264.7 cell line decreased TGF-beta secretion under BCG treatment (data not shown), we can discard this growth factor as a possible mediator of the effects of BCG on fibroblasts proliferation On the other hand we demostrated that RAW 264.7 activated by BCG produced FGF-2, which was able to induce fibroblast proliferation. The histological analyses from wounds treated with MACs-T-BCG plus L-NAME or 1400W revealed an increase in FGF-2 expression compared with MACs-T. Besides, BCG and L-NAME-treated tumors presented higher FGF-2 expression than untreated ones. The exact role of FGF-2 is controversial, since its function seems to depend on the relative concentration and the degree of activation in the specific tissue [35]. It has been reported that L-NAME inhibits the endothelial cell tube formation in response to FGF-2 [36]. Thus, we could speculate that the angiogenic activity in our model could be inhibited by L-NAME, and that only the fibrotic activity would persist. Interestingly, it has been reported that an anti-idiotypic strategy mimics the biological activity of FGF-2, inhibiting the progression of an experimental bladder cancer [37]. However, further experiments should be performed to clarify the precise role of FGF-2 in response to BCG in bladder cancer. Our present results seem to suggest that, at least in part, this factor was involved in the mechanisms of bladder cancer control by BCG.

**Figure 7:**
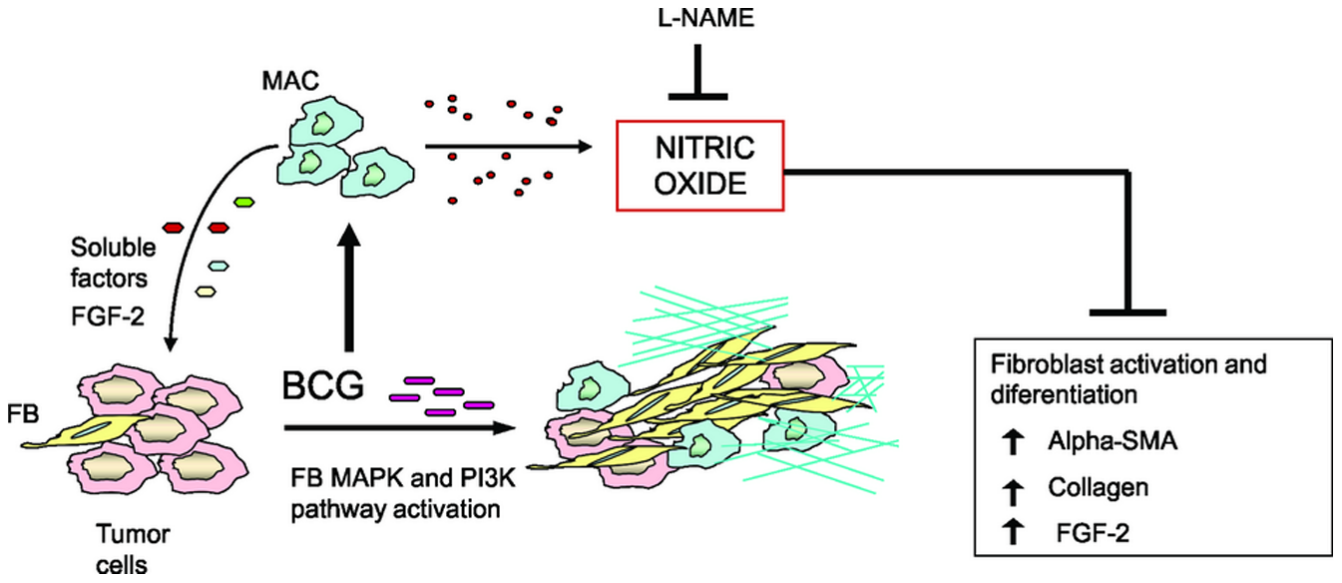
Combined therapy of BCG and L-NAME exerts an antitumoral effect through modulation of MACs and fibroblasts in the tumor microenvironment. BCG directly induces fibroblast proliferation via MAPK and PI3K signaling pathways as well as alpha-SMA and collagen expression. BCG induces in MACs the production of NO and soluble factors including FGF-2 which induce fibroblast proliferation. BCG therapy increases collagen deposition and expression of alpha-SMA and FGF-2 in bladder tumors The treatment with L-NAME, improves the stimulation of fibroblasts by BCG.

In conclusion, as shows figure 7, our findings suggest that when BCG is used as immunotherapy in bladder cancer, BCG targets not only immune or tumor cells, but also fibroblasts either directly and/or through activated MACs. We believe that NO produced by MACs and tumor cells, is an undesired effect of BCG immunotherapy and therefore, the possibility of modulating iNOS activity concomitantly with conventional BCG therapy in patients should be considered.

## Materials and Methods

### Cell Culture

NIH-3T3 (ATCC) cell line and macrophages RAW 264.7 were maintained in DMEM (Gibco®). The murine bladder cancer cell line MB49 was cultured in MEM (Gibco®). All cultured media were supplemented with 2 mM L-glutamine, 80 ug/ml gentamycin and 5-10% fetal calf serum (FCS) in a humidified atmosphere with 5% CO2.

### BCG

Living organisms of an attenuated strain of *mycobacterium tuberculosis*, (Pasteur 1173 P2 strain, were obtained from Instituto Nacional de Producción de Biológicos A.N.L.I.S. C.G. Malbrán, Buenos Aires, Argentina.

### Cell viability assay

5×10^3^ NIH-3T3/100 ul were cultured in 96-well plates (Greiner Labortechnik GmbH. Maybachstrabe 2, D-72636 Frickenhausen). After 24 h incubation, different concentrations of BCG from 1.5 to 7.5×10^6^ CFU/ml were added, and cells were cultured for 24 h. Cell viability was determined by the MTS assay (Promega). To evaluate if MTS assay correlate with cell proliferation, similar assays were made in 6 well dish and NIH-3T3 cell proliferation were determined by cell counting with Trypan Blue. To evaluate whether the MAPK or PI3K pathways were involved in BCG induction, NIH-3T3 cells were treated with BCG plus 10 and 20 µM of LY 294002 (Millipore) for 24 h or 10 and 50 uM of PD 98059 (Millipore) for 48 h. To evaluate NOS inhibitor activity on fibroblast viability, similar experiments were carried out in the presence of different concentrations of L-NAME (N-nitro-L-arginine-methyl-ester, Sigma). In other series of experiments 50% of conditioned media (CM), from peritoneal MACs from different groups described before, or from RAW 264.7 cells treated with BCG, were added during 48 h and cell viability was determined by MTS. When proliferation was measured by ^3^(H)-thymidine incorporation, CM was preincubated 1 h with 10 µg/ml of the blocking monoclonal anti FGF-2 antibody (DB3), or normal mouse IgG as a control. Control was carried out with exhausted culture media (from the same NIH-3T3 cells). 0.5 ng/ml of FGF-2 was used as a positive control. After 2 h, 1 µCi ^3^(H)thymidine/well was added and cells were incubated for 48 h. The assay was stopped with 50 µl/well of 6 M guanidinium chloride. Cells were lysed by three freeze-thaw cycles. The cellular DNA was collected on Whatman GFC filters using a harvester (Cell Harvester 8, Nunc), fixed with 96% ethanol, air dried and incorporated radioactivity was determined in the presence of 1 ml of scintillation solution (OptiPhos Hifase 3) in a liquid scintillation counter (Packard 1600 TR, Canberra Company).

### Peritoneal macrophages

Taking into account that in a mouse model the number of intratumoral macrophages is very low and that BCG triggers systemic effects being some of them correlated with response to treatment [38], in this work, we have chosen to use peritoneal macrophages since, the peritoneum is from where, we can obtain more number of cells per mouse.

C57BL/6J male mice (approximately 8 weeks old), were obtained from our Institute Animal Care Division. Animal care was followed in accordance with the international procedure for the Care and Use of Laboratory Animal. Protocols were approved by the Institutional Review Board.

MB49 cells (5×10^5^ cells in 0.1 ml), were s.c. injected into the left flank of syngeneic mice. BCG (6×10^6^ CFU/ml), was intratumorally injected twice a week for twenty days starting 24 hs after tumor cell inoculation. Control mice were equally injected with saline solution. L-NAME (0.2 g/kg mouse), was added to drinking water. After twenty days MACs were obtained by washing the peritoneal cavity with cool PBS-EDTA 0.02%. MAC had been purified from the peritoneal washes by plastic adhesion for 2 h. For in vivo assay of wound repair, 10^6^ MACs from each experimental group were placed onto wounds, n = 6.

### Preparation of the Conditioned Media (CM)

RAW 264.7 cell line was treated in vitro with BCG (3×106 CFU/ml) or L-NAME (2 mM) and peritoneal MACs were obtained from tumor-bearing mice treated in vivo with BCG (6×106 CFU/ml) or L-NAME (2 mM). After each treatment of semiconfluent RAW 264.7 cells or peritoneal MACs monolayer growing in 35 mm plates were extensively washed with PBS. Serum-free medium (1 ml), was added and incubation was continued for 24 hours. CM was harvested and the number of cells in the remaining monolayer was quantified. CM samples were aliquoted, stored at 80°C and used only once after thawing. After the exhaustive washing no traces of BCG were observed.

### NO production

NO production in MACs supernatants was determined after 24 h of BCG treatment either with or without the addition of L-NAME in the case of RAW 264.7 cells and directly, from peritoneal MACs of tumor bearing mice which had been treated with L-NAME using the protocol as previously described [11].

### Western blot assay

6×10^5^ NIH-3T3 cells were plated in 100-mm culture dishes. When the monolayer reached 80% of confluence, the cultures were treated with 3×10^6^ CFU/ml of BCG at the indicated times. Cells were gently washed with PBS and lysed using protein extraction lysis buffer (50 mM Tris-HCl (pH 8.0); 100 µM NaCl; 1% Triton, 1 µM/ml aprotinin, 1 mM phenylmethylsulfonyl fluoride, 2 µg/ml leupeptin and 10 mM EDTA/EGTA). Protein concentration was determined by Bradford method according to the manufacturer's instruction. Aliquots from the cell lysates were separated (50 µg for p-AKT or p-ERK and 80 µg for alpha-SMA, collagen I or FGF-2), and analysed in 10% sodium dodecyl sulfate-polyacrylamide gel (SDS-PAGE) and transferred onto a PVDF membrane. After blotting, the membrane was incubated with primary antibody (p-AKT, (sc-7985-R); AKT (sc-8312); p-ERK (sc-7383), ERK ½ (BD Biociences 610124), (1,1000), collagen type I (sc-8784); alpha-SMA (sc-53142) (1,200) or FGF-2 (1.25 ug/ml) developed by A. Baldi as described [39]) and incubated with horseradish peroxidase-conjugated secondary antibody (Millipore) (1,2000 dilution), for 1 h at room temperature. The blots were developed using the ECL detection kit (GE Healthcare, USA), and exposed to X-ray film. Bands were analysed in software. Then, membranes were stripped and incubated over with ERK or AKT antibodies respectively or beta-actin (Sigma, A5441) (1,20000 dilution), was used as a loading control. In the WB corresponded to FGF-2, 20 ng of purified murine FGF-2 was used as a positive control. Densitometric units of p-AKT or p-ERK were relativized to the correspondent band of AKT or ERK. Densitometric units of collagen, alpha-SMA or FGF-2 were relativized to the correspondent band of beta-actin or GAPDH. Values were referred as a fold change of control.

### Immunofluorescence assay

NIH-3T3 cells growing in chamber slides in complete medium were treated with BCG or CM from peritoneal MACs or from RAW264.7 cells as described before. Subconfluent monolayers were gently washed with cold saline buffer and processed for immunofluorescence. Slides were fixed with formalin 4% in PBS for 15 minutes and permeabilized. Nonspecific antibody binding was blocked with Tween-20 0.1% plus 2% succinimidyl 4-formylbenzoate in PBS for 60 minutes at room temperature.

Fixed cells were incubated overnight with rabbit polyclonal antibody (1∶100 dilution) anti-alpha-SMA (sc-53142), collagen I (sc-8784) or FGF-2 (2.5 ug/ml). Rabbit IgG was used as isotype control. Goat anti-rabbit IgG-fluorescein conjugate (Chemicon®; 1∶150 dilution) in PBS, served as the secondary antibody. Nuclei were counterstained with DAPI, slides were observed in an Nikon Eclipse™ E400 fluorescence microscope and photographed with a Coolpix® 995 digital camera at 400 magnification.

### Immunohistochemistry

Alpha-SMA and FGF-2 expression levels were histologically determined in paraffin-embedded sections in s.c. MB49 tumors from mice treated for 24 days and in wounds, by HRP immunohistochemical technique. Specific antibodies to alpha-SMA,(sc-53142, 1∶100 dilution) and FGF-2 (2.5 ug/ml) were used. Labelled streptavidin biotin (LSAB; Dako Cytomation, CA), at first biotinylated link universal was appplied. In a negative controls, primary antibody were omitted in each case. For collagen deposition determination, Masson's trichrome staining was performed as previously described (Carson F.L: Histotechnology. A self-instructional test. ASCP Press, Chicago, 1990.)

### Skin Wounds repair assay *in vivo*

6 to week old male C57BL/6J mice were anesthetized with an intraperitoneal injection of a combination of 70 mg/kg ketamine and 5 mg/kg xylazine, mice were and depilated in the dorsal zone. A 3 mm-diameter circle wound, including the skin and panniculus carnosus muscle were defined using scissors. The wounds were photographed, and then 1×10^6^ peritoneal MACs resuspended in 33 ul of PBS-glicerol, 2 mM L-NAME, or 1 µM 1400 W (Calbiochem), were placed onto the wound. The wound closure was photographed every day one week. At 24 h some mice were sacrificed, the wound was isolated, fixed and embedded in paraffin. Using Image J software, up to four perpendicular diameters (d) were measured. The surface of the wound was calculated using the following formula: 3.1416×(d/2)^2^ and the wound closure was calculated as the percentage of the initial wound area at day zero. We performed a new set of experiments where skin wounds were created in mice administered with L-NAME (0.2 g/kg mouse) as described [11].

### Ethics Statement

C57BL/6J male mice (approximately 8 weeks old), were obtained from our Institute Animal Care Division. Animal care was followed in accordance with the international procedure for the Care and Use of Laboratory Animal. Protocols were approved by the Institutional Review Board COMITE INSTITUCIONAL DE CUIDADO Y USO DE ANIMALES DE LABORATORIO “CICUAL”. Res (CD) 2079/07. Secretaría de Ciencia y Técnica FACULTAD DE MEDICINA, U.B.A. Paraguay 2155, Buenos Aires City, Argentina (C1121ABG).

### Statistical analysis

Three independent experiments were carried out and only one is shown. The results were expressed as the mean ± SD of six replicates per group. Statistically significant values were compared using ANOVA and Bonferroni's contrast by using Graph Pad InStat statistical package (version 3.01). p-values lower than 0.05, were considered statistically significant.
